# Widespread Distribution of Carbapenem-Resistant *Klebsiella* spp. in Clinical and Environmental Settings

**DOI:** 10.3390/antibiotics14111140

**Published:** 2025-11-10

**Authors:** Karla Vanessa Molina Maldonado, Julia Marchese Pereira, Tamires Nascimento da Costa, Gabriel Lemos Buss, Kethlen Natiele de Almeida Pereira, Anelise Baptista da Silva, Gertrudes Corção, Ândrea Celestino de Souza, Amanda Silva Martins, Diego Rodrigues Falci, Ariane Baptista Monteiro, Claudia Flores, Kayo Bianco, Maysa Mandetta Clementino, Carlos Alexandre Sanchez Ferreira, Renata Medina-Silva, Sílvia Dias de Oliveira

**Affiliations:** 1 Laboratório de Imunologia e Microbiologia, Escola de Ciências da Saúde e da Vida, Pontifícia Universidade Católica do Rio Grande do Sul, Porto Alegre 90619-900, RS, Brazil; vanessa.karla@edu.pucrs.br (K.V.M.M.); j.marchese@edu.pucrs.br (J.M.P.); nascimento.tamires@edu.pucrs.br (T.N.d.C.); anelise.baptista@gmail.com (A.B.d.S.); cferreira@pucrs.br (C.A.S.F.); renata.medina@pucrs.br (R.M.-S.); 2 Programa de Pós-Graduação em Biologia Celular e Molecular, Escola de Ciências da Saúde e da Vida, Pontifícia Universidade Católica do Rio Grande do Sul, Porto Alegre 90619-900, RS, Brazil; 3 Departamento de Microbiologia, Imunologia e Parasitologia, Instituto de Ciências Básicas da Saúde, Universidade Federal do Rio Grande do Sul, Porto Alegre 90035-003, RS, Brazil; corcao@ufrgs.br; 4 Laboratório Clínico, Hospital São Lucas, Pontifícia Universidade Católica do Rio Grande do Sul, Porto Alegre 90610-001, RS, Brazil; andrea.souza@pucrs.br (Â.C.d.S.); amanda.martins@pucrs.br (A.S.M.); 5 Serviço de Controle de Infecção, Hospital São Lucas, Pontifícia Universidade Católica do Rio Grande do Sul, Porto Alegre 90610-001, RS, Brazil; diego.falci@pucrs.br (D.R.F.); ariane.monteiro@pucrs.br (A.B.M.); 6 Núcleo de Formação Específica em Medicina Interna e Diagnóstica, Escola de Medicina, Pontifícia Universidade Católica do Rio Grande do Sul, Porto Alegre 90610-001, RS, Brazil; 7 Instituto Nacional de Controle de Qualidade em Saúde INCQS/FIOCRUZ, Rio de Janeiro 21040-900, RJ, Brazilkayo.bianco@fiocruz.br (K.B.); maysa.mandetta@fiocruz.br (M.M.C.)

**Keywords:** carbapenem-resistant *Klebsiella* spp., biofilm, antimicrobial resistance, healthcare-associated infection, polymyxin B

## Abstract

**Background/Objectives:** *Klebsiella* spp., particularly *K*. *pneumoniae*, are major opportunistic pathogens in healthcare settings driven by carbapenemase- and ESBL-producing strains. We assessed antimicrobial resistance and biofilm formation abilities in *Klebsiella* spp. from a Brazilian tertiary hospital and related environments and characterized capsular types. **Methods**: Over six months (July–December 2023), 303 carbapenem-resistant *Klebsiella* spp. were collected from clinical specimens (*n* = 198), ICU/non-ICU surfaces (*n* = 79), hospital sewage (*n* = 22), and stream water (*n* = 4). Species were identified by MALDI-TOF. Susceptibility testing covered eight antibiotic classes, focusing on carbapenems and polymyxin B. Biofilm formation was quantified by crystal violet, and capsular typing used *wzi*/K-locus approaches. **Results**: Most isolates (70.95%) had meropenem MICs ≥ 128 μg/mL, while 77.6% (*n* = 235) remained susceptible to polymyxin B. Resistance profiles largely consisted of extensive drug resistance (95.4%), with 1.3% exhibiting pandrug resistance, including isolates from bed rails. Biofilm formation was detected in 96.7% of isolates, mainly weak (67.6%) or moderate (28%), with 4.4% being strong producers. Among the *Klebsiella* isolates analyzed, 21 K types were identified with an uneven distribution dominated by K64, followed by K24, K173, and K50. K75 was the only K type detected across all sources—clinical isolates, bed-rail surfaces (non-ICU), wastewater, and fluvial water. **Conclusions**: Carbapenem-resistant *Klebsiella* spp. exhibited widespread resistance, with residual susceptibility to aminoglycosides, ceftazidime–avibactam, and polymyxins. Environmental reservoirs—hospital surfaces, sewage, and stream water—harbored resistant biofilm producers, reinforcing their role in persistence and dissemination. K-typing revealed concentrated distribution (predominantly K64) and cross-source K75. These findings underscore the urgency of integrated strategies combining molecular surveillance, antimicrobial stewardship, and environmental control.

## 1. Introduction

Antimicrobial resistance (AMR) constitutes one of the most critical global threats to public health, directly causing at least 1.27 million deaths and contributing to nearly 5 million deaths worldwide in 2019 [1]. Healthcare-associated infections (HAIs) remain a major concern across high-, middle-, and low-income settings, particularly as AMR burdens rise. In the WHO Region of the Americas (AMRO), an estimated 569,000 deaths in 2019 were associated with bacterial AMR, representing approximately 11.5% of global AMR-associated deaths [2]. According to the Pan American Health Organization (PAHO) Latin American Network for Antimicrobial Resistance Surveillance (ReLAVRA), non-susceptibility to carbapenems among Gram-negative bacilli in Latin America reached nearly 21% in 2016 (PAHO, 2018) [3], with some countries reporting 20–50% prevalence among multidrug-resistant Enterobacterales [4]. A multicenter surveillance across Latin America and the Caribbean (2015–2020) reported that 65.3% of carbapenemase detections occurred in carbapenem-resistant *Enterobacterales*, with *Klebsiella pneumoniae* representing 75.9% of isolates harboring *bla*_KPC_ [5].

The main routes of HAI transmission involve direct and indirect contact with contaminated clinical materials, surfaces, hands, or gloves [6]. Healthcare workers, patients, and visitors can acquire pathogens through contact with contaminated surfaces and subsequently transfer them to other sites or individuals within healthcare settings [7,8]. Additionally, biofilms—complex and structured microbial communities adherent to surfaces—represent a major challenge, since biofilm-associated cells exhibit increased tolerance to antibiotics, biocides, and desiccation compared to their planktonic counterparts [9,10]. These structures act as reservoirs enabling cross-transmission, particularly when cleaning and disinfection are suboptimal [11]. Approximately 65–80% of bacterial infections are biofilm-associated [12], and in *K. pneumoniae*, biofilm formation is influenced by multiple features, including capsular composition, which modulates adhesion, maturation, and the overall architecture of biofilms, thereby contributing to persistence in clinical settings [13]. Over 130 capsular (K) types have been identified in *K. pneumoniae* (https://bigsdb.pasteur.fr/klebsiella/ accessed on 10 October 2025), displaying structural diversity that affects virulence, immune evasion, and biofilm-related processes [13].

Beyond direct contact, hospital wastewater constitutes an important reservoir and dissemination pathway for multidrug-resistant (MDR) organisms [14,15]. Hospital effluents combine fecal matter, biological fluids, and chemical waste, carrying pathogenic bacteria and antimicrobial residues from clinical activities [14,16,17]. These effluents are enriched with resistance genes and mobile genetic elements (MGEs), supporting the persistence and exchange of resistance traits within microbial communities. Moreover, sub-inhibitory concentrations of antibiotics, disinfectants, and heavy metals impose continuous selective pressure, favoring the survival and adaptation of resistant bacteria [18]. As these effluents enter municipal wastewater systems, resistant bacteria, antibiotic residues, and genetic elements can reach surrounding aquatic environments, extending the dissemination of antimicrobial resistance beyond hospital boundaries [19]. Altogether, the coexistence of selective agents and dense microbial consortia in hospital sewage and downstream systems creates ideal conditions for horizontal gene transfer between environmental and clinically derived bacteria, reinforcing the One Health perspective of interconnected human, environmental, and microbial domains.

Infections caused by *Klebsiella* spp. are commonly treated with third- and fourth-generation cephalosporins, fluoroquinolones, or carbapenems [20]. However, *Klebsiella* spp. exhibit multiple resistance mechanisms, most notably the production of β-lactamases [21,22]. Extended-spectrum β-lactamases (ESBLs), which hydrolyze β-lactam antibiotics such as third- and fourth-generation cephalosporins and aztreonam, have markedly reduced the effectiveness of these drugs and driven the use of carbapenems as first-line therapy for severe infections [23]. The widespread and frequent use of carbapenems has, in turn, favored the emergence of carbapenem-resistant strains, including carbapenem-resistant *Enterobacterales* (CRE) [24]. Although carbapenems are stable to hydrolysis by ESBLs and AmpC β-lactamases, resistance has increasingly resulted from the production of carbapenemases—such as KPC, NDM, OXA-48-like, VIM, and IMP—which hydrolyze carbapenems and other β-lactams. Carbapenem resistance can be further enhanced by alterations or loss of porin channels (OmpK35 and OmpK36), reducing antibiotic influx, particularly when combined with ESBL or AmpC β-lactamase production [25,26]. Furthermore, plasmid-mediated AmpC β-lactamases (e.g., CMY-2 and DHA-1) confer resistance to expanded-spectrum cephalosporins and can potentiate carbapenem non-susceptibility when associated with porin loss [27,28].

The cumulative expression of these enzymes, alone or in combination, represents a major therapeutic challenge, greatly limiting the effectiveness of β-lactam agents against *Klebsiella* infections. As a result, polymyxins re-emerged as last-resort antibiotics for the treatment of CRE [29,30]. Polymyxins are cationic polypeptides that interact with the negatively charged lipid A component of lipopolysaccharides (LPS), causing outer membrane disruption and increased permeability [31]. However, resistance to polymyxins has been increasingly documented worldwide, arising mainly from chromosomal mutations that inactivate *mgrB* or activate the *PhoPQ* and *PmrAB* regulatory systems. These changes lead to lipid A modification through the addition of positively charged moieties such as 4-amino-4-deoxy-L-arabinose (L-Ara4N) or phosphoethanolamine, reducing the net negative surface charge and, consequently, polymyxin binding [32]. In addition, plasmid-borne *mcr* genes have facilitated horizontal transfer of polymyxin resistance across bacterial species and environmental reservoirs [33]. Non-LPS-dependent mechanisms—typically associated with lower levels of resistance—have also been reported, including increased production of anionic capsular polysaccharides in *Klebsiella* spp. [30].

Based on the aforementioned, this study aimed to characterize antimicrobial-resistant *Klebsiella* spp. isolated from different sources within and around a hospital environment, including clinical samples. Previous studies by the same research group identified bed rails as the predominant sites for bacterial persistence in hospital settings [34,35]. However, few studies have addressed the distribution of antimicrobial-resistant *Klebsiella* spp. in hospital wastewater and their potential dissemination into water streams. Therefore, we compared the antimicrobial resistance, biofilm formation, and capsular typing profiles of isolates recovered from ICU and non-ICU bed rails with those obtained from hospital wastewater. The same features were analyzed in clinical isolates collected from patients during the same period. In addition, isolates recovered from a nearby water stream that was not connected to the hospital wastewater were included for environmental comparison.

## 2. Results

### 2.1. Sample Collection, Detection Rates, and Species Identification

During the study period, 504 samples were collected from bed rails in adult ICU and non-ICU units (*n* = 240 each), with additional samples from hospital wastewater (*n* = 12) and a nearby stream (*n* = 12). Overall, 206 bacterial isolates were recovered (75.7% bed rails, 20.4% wastewater, 3.9% stream), of which *Klebsiella* spp. accounted for 51% (105/206) and were detected across all sources.

In parallel, the hospital clinical laboratory provided a patient-derived set of carbapenem-resistant *Klebsiella* spp. collected during the same six-month period. Combining this clinical set with the *Klebsiella* spp. recovered from bed rail and water samples yielded a total of 303 carbapenem-resistant *Klebsiella* spp.: 198 from the clinical laboratory, 62 from ICU bed rails, 17 from non-ICU bed rails, 22 from wastewater, and 4 from stream water. The isolates were identified by MALDI-TOF as *K. pneumoniae* (*n* = 295), *K. oxytoca* (*n* = 7), and *K. variicola* (*n* = 1).

### 2.2. Antimicrobial Susceptibility

**Figure 1:**
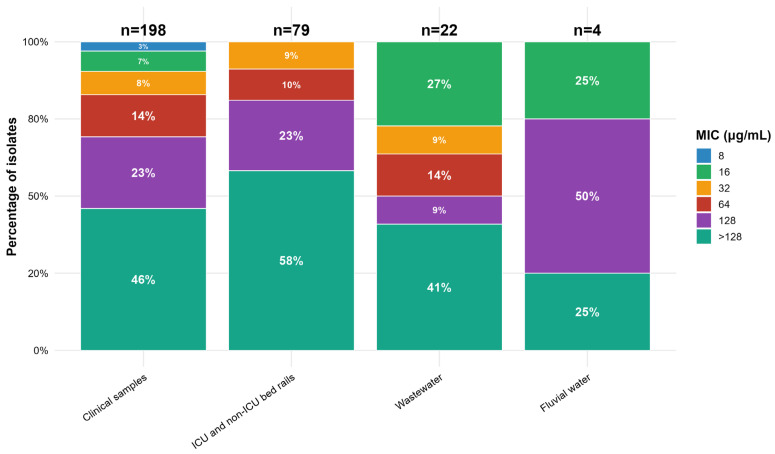
Stacked bar chart of meropenem minimum inhibitory concentration (MIC) categories (8, 16, 32, 64, 128, >128 µg/mL) across *Klebsiella* spp. isolated from clinical samples, ICU/non-ICU bed rails, wastewater, and fluvial water. Sample sizes (n) are shown above each bar, and segment labels indicate within-source percentages.

All presumptive *Klebsiella* colonies recovered on meropenem-supplemented MacConkey agar were confirmed as meropenem-resistant by MIC. Phenotypic and typing analyses were then performed exclusively on these isolates. Most isolates (70.95%) had MIC values for meropenem ≥ 128 μg/mL, and high meropenem MIC values were found regardless of origin (Figure 1). The MIC_50_ and MIC_90_ for meropenem in clinical sample isolates were 128 and > 128 μg/mL, respectively. In bed rail sample isolates, the MIC_50_ and MIC_90_ values were both > 128 μg/mL.

The majority of *Klebsiella* spp. isolates were susceptible to polymyxin B (*n* = 235, 77.6%). The polymyxin B resistance rates found in the different sources were 41.8% of bed rail isolates (*n* = 33), 25% of stream isolates (*n* = 1), 15.1% of clinical isolates (*n* = 30), and 18.2% of hospital wastewater isolates (*n* = 4) (Supplementary Table S1). A significantly higher proportion of polymyxin B-resistant *Klebsiella* spp. isolates was observed among samples from bed rails compared to other sources (*p* = 0.0007). Pairwise comparisons confirmed that the proportion of isolates resistant to polymyxin B was significantly higher in samples from bed rails compared to those from clinical samples (adjusted *p* = 0.027) and wastewater samples (adjusted *p* = 0.006). Among the clinical isolates, MIC_50_ and MIC_90_ values for polymyxin B were ≤1 and 8 μg/mL, respectively, whereas 2 and 16 μg/mL were found for the bed rails isolates, respectively.

**Figure 2:**
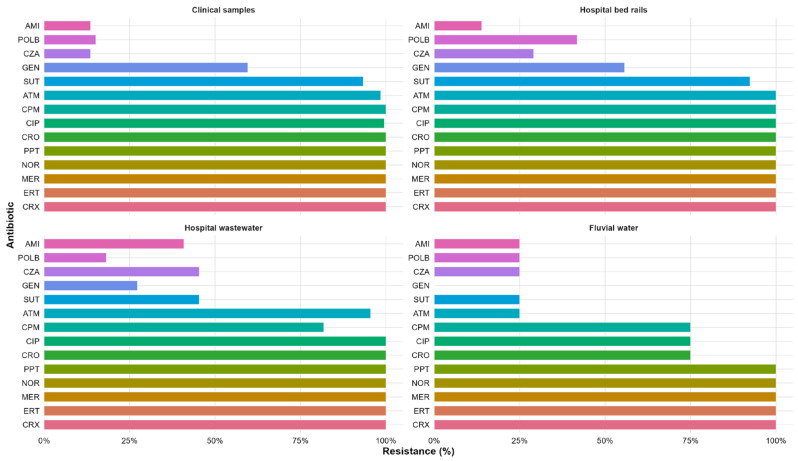
Grouped bar chart showing the percentages of antimicrobial resistance among *Klebsiella* spp. isolates by antibiotic and source. The antibiotics tested were cefepime (CPM), cefuroxime (CRX), ceftriaxone (CRO), aztreonam (ATM), ertapenem (ERT), meropenem (MER), gentamicin (GEN), amikacin (AMI), ciprofloxacin (CIP), norfloxacin (NOR), sulfamethoxazole-trimethoprim (SUT), ceftazidime-avibactam (CZA), piperacillin-tazobactam (PPT), and polymyxin B (POLB).

All carbapenem-resistant *Klebsiella* spp. isolates were also tested for susceptibility to seven other classes of antibiotics using the disk diffusion method. All clinical isolates were also resistant to cephalosporins, piperacillin-tazobactam, and norfloxacin. High resistance rates were found for ciprofloxacin (99.5%), aztreonam (98.5%), and sulfamethoxazole-trimethoprim (93.4%). Resistance rates were 59.6% for gentamicin, 25.8% for ceftazidime-avibactam, and 13.6% for amikacin (Figure 2). Resistance was significantly lower to amikacin, ceftazidime-avibactam, and gentamicin among the isolates from clinical simples (*p* < 0.0001).

The highest resistance rates were found among isolates from bed rails. All isolates were resistant to the tested β-lactams and fluoroquinolones, as well as piperacillin-tazobactam. The other resistance rates found were 92.4% for sulfamethoxazole-trimethoprim, 55.7% for gentamicin, 29.1% for ceftazidime-avibactam, and 13.9% for amikacin (Figure 2). Similar to the isolates from clinical samples, isolates from bed rails showed significantly lower resistance to gentamicin, amikacin, and ceftazidime-avibactam (*p* < 0.0001).

All the isolates from hospital wastewater were resistant to fluoroquinolones and piperacillin-tazobactam, and none were susceptible to β-lactams. The resistance rates to other antibiotics were as follows: 45.4% for sulfamethoxazole-trimethoprim and ceftazidime-avibactam, 40.9% for amikacin, and 27.3% for gentamicin (Figure 2). Resistance rates to gentamicin, amikacin, ceftazidime-avibactam and sulfamethoxazole-trimethoprim were significantly lower when compared to several other antibiotics in the isolates from wastewater (*p* < 0.05). Wastewater isolates showed significantly lower resistance rates to gentamicin and sulfamethoxazole-trimethoprim compared with clinical and bed rail isolates (*p* < 0.05). In contrast, they displayed significantly higher resistance rates to amikacin (*p* < 0.05).

Unlike the other sources, isolates from the stream water samples were susceptible to cephalosporins, and 50% were also susceptible to aztreonam. However, all isolates were resistant to carbapenems, norfloxacin, and piperacillin-tazobactam. Most isolates (75%) were resistant to ciprofloxacin, in contrast to 25% that were resistant to amikacin, sulfamethoxazole-trimethoprim, and ceftazidime-avibactam. None of the isolates were resistant to gentamicin (Figure 2).

All *Klebsiella* spp. isolates were classified considering their resistance profile according to the Magiorakos et al. [36]. Most isolates (95.4%) were classified as XDR (*n* = 289), 3.3% as MDR (*n* = 10), and 1.3% as PDR (*n* = 4). The PDR isolates were obtained from bed rails in the ICU (*n* = 3) and wastewater (*n* = 1). Detailed results of antimicrobial susceptibility testing are presented in Table S1 of the Supplementary Materials.

### 2.3. Biofilm Formation

**Figure 3:**
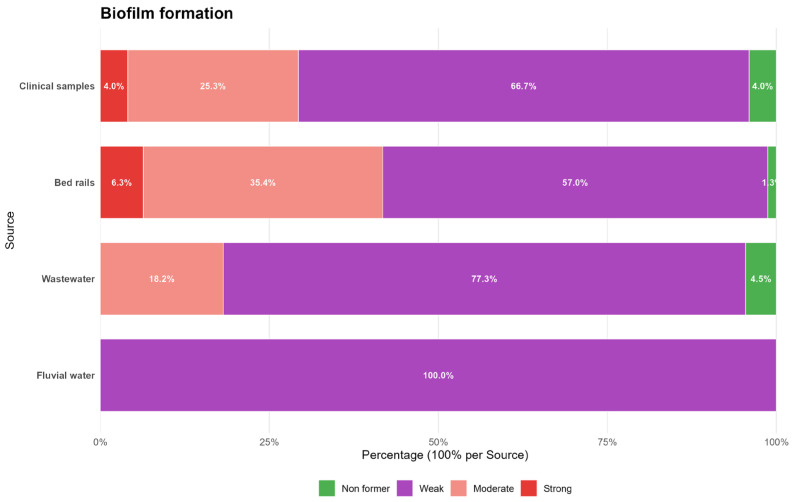
Distribution of biofilm phenotypes (non-former, weak, moderate, strong) among *Klebsiella spp.* isolates from clinical samples, bed rails, hospital wastewater, and fluvial water, displayed as a 100% stacked bar chart normalized within each source.

The bacterial isolates were analyzed for their ability to form biofilm. Among them, 3.3% (*n* = 10) were classified as non-biofilm formers, 65.3% (*n* = 198) as weak formers, 27.1% (*n* = 82) as moderate formers, and 4.3% (*n* = 13) as strong formers (Figure 3). No statistically significant association was observed between the biofilm formation classification and the source of *Klebsiella* spp. isolates (*p* = 0.092) or polymyxin B resistance among *Klebsiella* spp. isolates (*p* = 0.990).

### 2.4. K Typing

**Figure 4:**
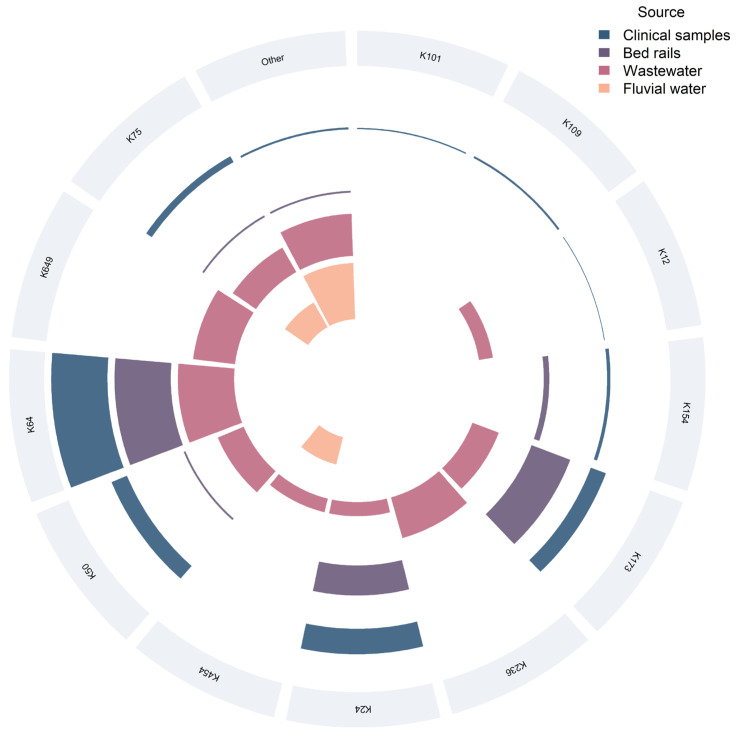
Circular stacked chart showing capsular K-type distribution by source. Each labeled sector represents a K type; colored arcs indicate contributions from clinical samples, bed rails, hospital wastewater, and fluvial water. Arc length is proportional to the number of isolates per K type and source.

A total of 21 distinct K types was identified among the 295 *Klebsiella* (294 *K*. *pneumoniae* and 1 *K*. *variicola*) isolates analyzed (Figure 4). The overall distribution was highly uneven, with a marked predominance of K64 (40%), followed by K24 (18.6%), K173 (16.3%), and K50 (9.5%). Together, these four types accounted for approximately 84% of all isolates. Less common K types comprised K75 (4.75%) and K154 (2.7%); K109, K236, and K649 (1.0% each); K101, K12, K269, K3, and K167 (0.7% each); and K225, K454, K579, K66, K258, K359, and K925 (0.3% each).

Among clinical isolates, 12 distinct K types were observed. K64 was most prevalent (*n* = 84; 42.9%), followed by K24 (*n* = 38; 19.4%), K50 (*n* = 25; 12.8%), K173 (*n* = 24; 12.2%), and K75 (*n* = 10; 5.1%). Less frequent types included K154 (*n* = 5; 2.55%), K109 (*n* = 3; 1.5%), and K101, K12, K27, K330, and K66 (≤1.0% each).

In bed-rail isolates (ICU and non-ICU combined), K64 was dominant (*n* = 30), followed by K173 (*n* = 22), K24 (*n* = 16), K154 (*n* = 3), K50, K75 and K 167 (*n* = 1 each). When analyzed separately, ICU surfaces (*n* = 59) revealed six distinct K types, with K64 and K173 being co-dominant (*n* = 21 each; 35.6%), followed by K24 (*n* = 14; 23.7%); K50, K154, and K167 occurred at lower frequencies (*n* = 1 each). In non-ICU areas (*n* = 15), K64 predominated (*n* = 9; 60%), whereas K24 and K154 (*n* = 2 each) and K75 and K173 (*n* = 1 each) were less frequent.

Among wastewater isolates, 12 K types were detected; K64, K236 and K649 were most frequent, whereas K173, K50, K75, K12, K24, K269, K301, K454 and K505 were less common. In fluvial water, four K types—K75, K225, K454, and K579—were identified.

Notably, K75 was the only K type found across all sources, including clinical isolates, bed-rail surfaces (non-ICU), wastewater, and fluvial water.

Polymyxin B resistance mirrored the overall K-type distribution—led by K64 (*n* = 27; 40.9%), followed by K24 (*n* = 16; 24.2%), K50 (*n* = 8; 12.1%), and K173 (*n* = 7; 10.6%)—with resistant phenotypes rare in other K types. No significant association between K type and polymyxin B resistance was detected (χ^2^(20) = 18.907, *p* = 0.528). All four PDR isolates belonged to different K types: two K24, one K50, and one K173.

Regarding biofilm formation, weak producers were most frequent, followed by moderate, strong, and non-formers. Within the weak biofilm group, K64 predominated (*n* = 74; 39.8%), followed by K24 (*n* = 37; 19.9%) and K173 (*n* = 29; 15.6%). Among moderate biofilm formers, the same K types were most represented—K64 (*n* = 39; 48.75%), K173 (*n* = 15; 18.75%), and K24 (*n* = 10; 12.5%). Strong producers were less frequent but showed a similar pattern (K64, K24, and K173, each 23.1%; *n* = 3), whereas non-formers were scarce, with K24 (*n* = 4; 50%) being the most common. A chi-square test of independence indicated a global association between K type and biofilm category (χ^2^(60) = 93.116, *p* = 0.003956); however, no pairwise differences remained significant after Bonferroni correction. Overall, again, the frequency pattern mirrored the overall K-type distribution in the collection, with no evidence of enrichment of any single K type in a specific biofilm category. 

## 3. Discussion

All *Klebsiella* spp. isolates analyzed in this study were carbapenem-resistant and, in most cases, exhibited XDR phenotypes, with a few PDR isolates identified according to the criteria of Magiorakos et al. [36]. This resistance profile spanned multiple classes of both β-lactam and non-β-lactam agents, reflecting constrained therapeutic options and the potential for persistence and dissemination within healthcare environments. These findings align with worldwide surveillance data documenting the rising prevalence of carbapenem-resistant *Klebsiella pneumoniae* (CRKP) in both high- and middle-income countries [5,37,38]. In Brazil, resistance trends have been equally concerning, with carbapenem resistance escalating from below 10% in 2011 to over 30% in 2015, alongside a rise in polymyxin B resistance from 0% to 27.1% [39].

Phenotypically, we observed nearly universal resistance to β-lactams, including third- and fourth-generation cephalosporins and aztreonam, consistent with studies attributing such patterns to co-production of carbapenemases and ESBLs [40,41]. High fluoroquinolone resistance was also observed, in agreement with previous investigations that associate these phenotypes with mutations in *gyrA* and *parC* as well as plasmid-mediated *qnr* determinants [42,43]. Our study likewise showed almost complete resistance to trimethoprim–sulfamethoxazole (STX) and piperacillin–tazobactam (PPT), a finding comparable to global CRE reports. Residual susceptibility to amikacin, gentamicin, and ceftazidime–avibactam (CZA) was detected, corroborating recent evidence that these remain among the few active options against certain carbapenemase-producing strains [44,45,46]. Polymyxin B also retained activity in a substantial proportion of isolates, supporting its continued role as last-resort agent, provided its use is judicious and closely monitored for toxicity [47]. Nevertheless, the high proportion of polymyxin B-resistant isolates recovered from hospital surfaces (41.8%), particularly from ICU beds, remains a matter of special concern. These findings are consistent with Brazilian and international evidence demonstrating persistence of resistant isolates on inanimate surfaces [48,49]. Environmental persistence is further supported by studies where disinfection reduced microbial load in only a minority of sites, emphasizing the resilience of hospital-associated Gram-negative bacteria [50]. Expanding on these observations, selective pressure from disinfectants such as chlorhexidine may select for decreased susceptibility and co-select plasmid-borne resistance determinants among Gram-negative bacteria [51].

Collectively, our data demonstrate that environmental reservoirs of multidrug-resistant bacteria extend beyond patients and invasive devices, encompassing inanimate surfaces that silently sustain transmission. The effectiveness of disinfection depends on biocide choice, application method, and capacity to disrupt biofilms. Evidence indicates that conventional cleaning may be insufficient, highlighting the importance of integrating environmental surveillance, staff training, and targeted interventions into infection control programs [50]. In response to the detection of XDR isolates on bed rails, the hospital strengthened both terminal and routine cleaning protocols, upgraded disinfection barriers for high-touch surfaces, and expanded environmental microbiological surveillance for continuous monitoring. In parallel, contact precaution workflows, staff training, and antimicrobial management were revised—with an emphasis on stewardship—to reduce selective pressure and prevent the persistence of resistant clones in the care environment.

Our analysis also underscores the critical role of biofilm formation in bacterial persistence and resistance dissemination. Biofilms facilitate survival on inanimate surfaces despite cleaning and provide niches for horizontal gene transfer (HGT) [52,53]. Notably, *K. pneumoniae* biofilms can survive on dry surfaces for up to four weeks, further underscoring their role in persistence and transmission within healthcare environments [54]. In our series, 96.7% of carbapenem-resistant *Klebsiella* spp. isolates produced biofilms. Compared with previous reports, our isolates showed a higher proportion of weak producers, though even low-density biofilms can act as reservoirs for HGT [55,56]. Importantly, the biofilm matrix enhances proximity between cells and promotes plasmid transfer [57,58]. Within biofilm communities, additional mechanisms such as outer membrane vesicles and persister cells further enhance genetic exchange and long-term survival of *K. pneumoniae* under antimicrobial stress [57,59]. Mixed-species biofilms amplify these effects, enabling plasmid-mediated transfer of carbapenemase and colistin resistance genes across Enterobacterales [60]. Therefore, the high prevalence of biofilm producers in our study indicates a strong potential for persistence and genetic dissemination among multidrug-resistant *Klebsiella* spp., encompassing but not limited to carbapenem- and polymyxin-resistant strains. Additionally, other characteristics, such as capsular composition and production level, may interact synergistically to enhance environmental survival and spread [13].

Capsular typing provided valuable insight into the distribution and characteristics of carbapenem-resistant *K. pneumoniae* isolates recovered from clinical and environmental sources. In our dataset, four capsular types (K64, K24, K173, and K50) together accounted for 84.4% of all isolates, being detected in clinical samples, bed rails, and wastewater. Notably, K75 was the only K type identified in all sources, although without apparent association with hypervirulence or epidemiological predominance. K64 was the most frequent capsular type, consistent with its recurrent identification in hospital-associated carbapenem-resistant lineages, often linked to ST11 [61,62]. K24 was also detected in both clinical and environmental isolates, supporting its persistence across different settings [63,64]. By contrast, K173 and K50 were found less frequently and have been only sparsely reported in surveillance studies, suggesting either limited dissemination or regional occurrence. The recurrence of K64 and K24 in hospital-related isolates, together with the detection of K75 across all sampled sources, indicates continuous interconnection between clinical and environmental reservoirs. Therefore, the data presented herein is consistent with the persistence of specific capsular lineages under variable selective pressures, highlighting the importance of antimicrobial resistance stewardship within a One Health framework.

Our findings also revealed marked differences between isolates from surfaces, patients, and hospital sewage. Bacteria from the intrahospital environment showed higher resistance to aminoglycosides and STX than those from sewage, reflecting the stronger selective pressure of clinical settings where broad-spectrum antimicrobials are heavily used [65,66]. Sewage-derived isolates displayed lower resistance, consistent with wastewater studies reporting low gentamicin resistance in *E. coli* [67] and higher aminoglycoside susceptibility in Enterobacterales [68]. These observations are consistent with the influence of microbial community interactions and selective conditions shaping AMR persistence beyond antibiotic exposure alone [69]. Consequently, ARGs may accumulate and persist in sewage environments even when phenotypic resistance among isolated bacteria remains low. Nonetheless, selective forces in wastewater cannot be dismissed: antibiotic residues, heavy metals, and disinfectants shape microbial communities and may favor resistance maintenance, especially within biofilms [70]. Accordingly, hospital sewage consistently carries higher loads of resistant bacteria and ARGs than other sources [71,72].

Extending beyond hospitals, we also examined carbapenem-resistant *Klebsiella* spp. from stream waters, which comprised fewer isolates and exhibited a heterogeneous resistance profile: uniform carbapenem resistance, consistent gentamicin susceptibility, and variable cephalosporin resistance patterns. The stream evaluated, although not directly connected to hospital effluents, is impacted by urban discharges [73]. Fluvial environments thus act as dynamic reservoirs integrating resistomes from multiple anthropogenic sources [74,75]. Urban streams can amplify ARG dissemination into the broader environment and nearby populations [76,77]. The detection of carbapenem-resistant isolates with preserved polymyxin susceptibility is consistent with Brazilian environmental data where polymyxin resistance was rare [68]. Sites with higher organic contamination, including fecal-origin material, exhibited clinically relevant carbapenemases, suggesting that heterogeneous resistance profiles may derive partly from HGT involving environmental bacteria [78].

Methodologically, the crystal violet assay measures total biomass of biofilms without assessing cell viability or architecture. Variations in washing, fixation, cut-offs, and microtiter conditions may alter classifications and explain inter-laboratory variability [79,80]. In addition, since the assay was performed on polystyrene microplates under standardized laboratory conditions, it reflects the potential for biofilm formation rather than surface-specific behavior that may occur on clinical materials such as stainless steel or medical-grade plastics. Even considering these potential constraints, biofilms nonetheless emerged as a key mechanism of carbapenem-resistant *Klebsiella* persistence and dissemination, with 96.7% of isolates identified as biofilm producers, predominantly weak to moderate. While Rahdar et al. reported an association between stronger biofilm formation and resistance [81], our results align with Cusumano et al. [82] and Zheng et al. [83], showing that biofilm intensity does not universally predict resistance. Although no association was observed with polymyxin B, the high prevalence of ability to form biofilm among XDR strains suggests an important role in antimicrobial resistance persistence. This observation is consistent with reports emphasizing that biofilm–resistance relationships are antibiotic-specific and clonal-context dependent rather than universal traits [84].

## 4. Materials and Methods

### 4.1. Clinical Setting

The study was conducted over six months in a 335-bed tertiary care university teaching hospital located in the southern Brazil, with a total area of approximately 61,000 m^2^ and a 59-bed ICUs. In front of the hospital, there is a stream with a length of 17,830 m (550 m from the source of the stream) [85], and there are no drainage or pipeline connections linking the hospital to this stream.

### 4.2. Sampling in the Hospital Environment

**Figure 5:**
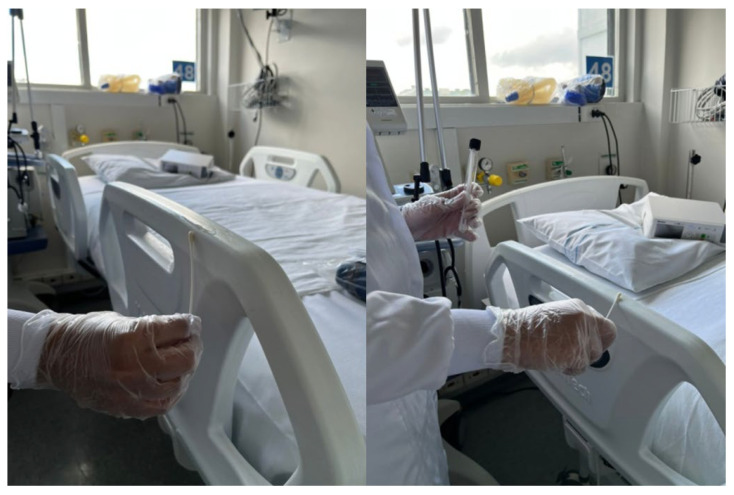
Representative images of the sample collection performed on the entire surface of the bed rails.

Samples were collected weekly from the bed rails of general adult ICU and non-ICU units. The entire lateral surface of the bed was sampled with a swab moistened with 0.85% saline (Labsynth, Diadema, Brazil) at least 3 h after the disinfection of the site (Figure 5). The swabs were placed in 0.85% saline and transported immediately to the laboratory. Following this, the swabs were inoculated in Brain Heart Infusion (BHI) broth (Kasvi, Madrid, Spain) with meropenem (1 μg/mL) (cat. Y0001252, European Directorate for the Quality of Medicines & Healthcare, Strasbourg, France) and subsequently incubated at 36 ± 1 °C for 12–18 h. BHI cultures were streaked on MacConkey agar (Kasvi, Madrid, Spain) with meropenem (1 μg/mL) and were incubated at 36 ± 1 °C for 18–24 h. Isolates showing Triple Sugar Iron (TSI) (Kasvi, Madrid, Spain) profiles compatible with *Klebsiella* spp. were stored at −80 °C in skim milk with 10% glycerol for further identification by matrix-assisted laser desorption/ionization time-of-flight (MALDI-TOF).

### 4.3. Water Sample Collections

During the same period, water samples were collected every two weeks, from the hospital domestic sewage (untreated influent) and from the stream located in front of the hospital. The hospital sewage samples were taken from a dedicated hospital sewer line manhole, prior to its junction with the municipal sewer. At each sampling point, a 50 mL water sample was collected aseptically into sterile glass vial. The collected samples were transported to the laboratory and then filtered using a vacuum pump with a 0.22 μm membrane (Sartorius, Göttingen, Germany). The membrane was placed in a sterile tube containing 10 mL of 0.85% saline for vortexing and release of microbial cells. Serial dilutions were made up to 10^−3^, considering the tube with the 0.22 μm membrane as the first dilution. Then, 100 μL of each dilution was spread on the surface of MacConkey agar with meropenem (1 μg/mL) using a Drigaslski loop. Colonies with a biochemical profile compatible with *Klebsiella* spp. were included in this study and stored at −80 °C in skim milk with 10% glycerol for subsequent identification by MALDI-TOF.

### 4.4. Clinical Isolates

The hospital laboratory provided the carbapenem-resistant *Klebsiella* spp. isolated from hospitalized patients and those from surveillance samples collected on patient admission during the same six months. Only one isolate per patient was included. The isolates were obtained from the following clinical specimens: urine (47%), tracheal aspirate (21.7%), blood culture (15.7%), abdominal fluid (3.5%), sputum (3.1%), drainage fluid (2.5%), surgical wound (2%), catheter (1%), and others (3.5%). The laboratory identified the isolates using the BD Phoenix M50 system (Becton Dickinson, Franklin Lakes, NJ, USA) and determined the antibiotic susceptibility by disk diffusion according to the guidelines of the Brazilian Committee on Antimicrobial Susceptibility (BrCAST) (https://brcast.org.br/wp-content/uploads/2022/09/Tabela-pontos-de-corte-clinico-BrCAST-01-02-2025.pdf, accessed on 1 February 2025) and the European Committee on Antimicrobial Susceptibility Testing (EUCAST) version 15.0 (valid from 1 January 2025). The isolates were stored at −80 °C in skim milk with 10% glycerol for further confirmation by MALDI-TOF.

### 4.5. Identification of Bacterial Isolates

Bacterial isolates that showed biochemical profiles compatible with *Klebsiella* spp. from all sampled sites were identified by MALDI-TOF mass spectrometry (Microflex LT, Bruker Daltonik^®^, Bremen, Germany). Bacterial isolates were cultured on BHI agar (Kasvi, Spain) and incubated at 37 °C for 18 to 24 h. Subsequently, 1 mL of the bacterial suspension was centrifuged at 5000 rpm for 5 min. The resulting pellet was treated with 70% ethanol and centrifuged again at 13,000 rpm for 2 min. The pellet was then transferred onto a stainless-steel MALDI target plate and overlaid with a matrix solution consisting of α-cyano-4-hydroxycinnamic acid (10 mg/mL in 50% acetonitrile and 2.5% trifluoroacetic acid). Each bacterial isolate was spotted in triplicate, and three independent readings were performed per sample. Protein spectra were acquired using FlexControl 3.3 software (Bruker Daltonik) with the MTB_autoX acquisition method, covering a mass range of 2 to 20 kDa. Spectral analysis was conducted using BioTyper 3.0 software (Bruker Daltonik^®^) by comparing the obtained profiles to those stored in the system’s reference database. Interpretation of identification scores followed the manufacturer’s criteria: scores ≥2.0 were considered reliable for species-level identification, while scores between 1.7 and <2.0 were accepted for genus-level assignments only. Each bacterial isolate was spotted in triplicate, and three independent readings were performed per sample.

### 4.6. Antimicrobial Susceptibility Testing

The minimum inhibitory concentration (MIC) of meropenem was determined by cation-adjusted broth microdilution according to the guidelines of the BrCAST and EUCAST, using a concentration range of 0.5 to 128 μg/mL. MIC assays were performed in triplicate for each *Klebsiella* spp. isolate. *Escherichia coli* ATCC 25922 was used as a quality control strain. In addition, a positive control with Mueller Hinton broth (Kasvi, Madrid, Spain) and 1 μL of bacterial dilution was made for each isolate without the addition of the antimicrobial. Also, a negative control was included for each plate using the Mueller Hinton broth culture medium only. The MIC_50_ and MIC_90_ values were calculated for the isolates from clinical samples and bed rails.

The isolates not susceptible to meropenem were characterized regarding susceptibility to other antibiotics. The MIC values for polymyxin B (cat. Y0000355, European Directorate for the Quality of Medicines & Healthcare, Strasbourg, France) were determined in the range of 0.5 to 32 μg/mL using the same conditions as above. The disk diffusion method according to EUCAST guidelines was used to evaluate the susceptibility to the following antibiotics: cefepime (CPM), cefuroxime (CRX), ceftriaxone (CRO), aztreonam (ATM), ertapenem (ERT), meropenem (MER), gentamicin (GEN), amikacin (AMI), ciprofloxacin (CIP), norfloxacin (NOR), sulfamethoxazole-trimethoprim (SUT), piperacillin-tazobactam (PPT), and ceftazidime-avibactam (CZA). *Escherichia coli* ATCC 25922 was used as a reference strain for quality control during the analyses, and the results were interpreted according to the criteria of the EUCAST guidelines. Isolates were classified as multidrug-resistant (MDR), extensively resistant (XDR) or pan-resistant (PDR) according to the classification described by Margiorakos et al. [36].

### 4.7. Determination of the Ability to Form Biofilm

The ability to form biofilm in polystyrene microplates was determined by the violet crystal method [86]. Each *Klebsiella* spp. isolate was grown in Lysogeny Broth (LB) (Kasvi, Madrid, Spain) overnight and then diluted to approximately 10^6^ colony forming units. Subsequently, 1 µL of this dilution was inoculated into each well containing 200 µL of LB and incubated at 36 ± 1 °C for 48 h. To eliminate planktonic cells, the wells were then washed twice with 200 μL of a phosphate-buffered saline (PBS) solution [8 g/L NaCl, 1.44 g/L Na_2_HPO_4_, 0.24 g/L KH_2_PO_4_ and 0.2 g/L KCl]. The microplates were dried at 60 °C for 15 min and then stained with 0.1% violet crystal for 5 min. After staining, the dye was removed, the wells were washed twice with 200 µL of PBS, and the microplate was incubated at 60 °C for 1 h. Subsequently, crystal violet-stained biofilms were solubilized with absolute ethanol for 15 min at room temperature, and the biomass was quantified at an optical density (OD) of 570 using a SpectraMax 190 microplate reader (Molecular Devices, San Jose, CA, USA) [87].

*Klebsiella* spp. isolates were classified as non-biofilm formers (OD ≤ OD_c_), weak biofilm formers (OD_c_ < OD ≤ 2OD_c_), moderate biofilm formers (2OD_c_ < OD ≤ 4OD_c_), and strong biofilm formers (4OD_c_ < OD) according to Stepanovic et al. [87]. The cut-off OD (ODc) is the mean OD plus three times the standard deviation of the negative control. For this procedure, the *Salmonella* Typhimurium ATCC 14028 strain, classified as a weak biofilm-forming strain, was used as the positive control and LB broth as the negative control.

### 4.8. Capsular Typing

*Klebsiella pneumoniae* and *K. variicola* isolates were subjected to capsular typing through *wzi* gene sequencing, following the protocol described by Brisse et al. (2013) [88]. The *wzi* gene encodes an outer membrane protein involved in capsule anchoring, whose sequence variability allows discrimination among different capsular types (K types).

Genomic DNA was purified using the PureLink Genomic DNA Mini Kit (Thermo Fisher Scientific, Waltham, MA, USA), according to the manufacturer’s instructions. DNA quality and concentration were assessed with a NanoDrop 2000C spectrophotometer (NanoDrop Technologies, Wilmington, DE, USA). All DNA samples were normalized to a final concentration of 10 ng/μL and stored at −20 °C until use. A fragment of approximately 580 bp of the *wzi* gene was amplified by PCR using the primers wzi_for2 (5′-GTG CCG CGA GCG CTT TCT ATC TTG GTA TTC C-3′) and wzi_rev (5′-GAG AGC CAC TGG TTC CAG AA[C/T] TT[C/G] ACC GC-3′). Each 25 μL reaction contained 0.2 μM of each primer, 1× GoTaq G2 Master Mix (Promega, Madison, WI, USA), 20 ng of template DNA, and nuclease-free water (Ambion, Austin, TX, USA) to complete the volume. PCR cycling conditions consisted of an initial denaturation at 94 °C for 2 min, followed by 30 cycles of 94 °C for 30 s, 55 °C for 40 s, and 72 °C for 30 s, with a final extension at 72 °C for 5 min.

PCR products were separated by electrophoresis in a 1.5% agarose gel for 1 h at 60 V in 1× Tris-acetate-EDTA (TAE) buffer and stained with GelRed (Biotium, Fremont, CA, USA). A 100 bp DNA ladder (Invitrogen, Carlsbad, CA, USA) was used as a molecular mass marker. DNA bands were visualized using an L-Pix Touch Transilluminator (Loccus Biotecnologia, Cotia, Brazil).

Amplicons were sequenced in both forward and reverse directions, in duplicate, using the BigDye Terminator kit and capillary electrophoresis on a SeqStudio sequencer (Thermo Fisher Scientific) at INCQS/FIOCRUZ (Rio de Janeiro, Brazil). Chromatogram quality was evaluated with SeqScape 4.0 software (Thermo Fisher Scientific). The resulting sequences were submitted to the *Klebsiella* Pasteur MLST database (http://bigsdb.pasteur.fr/klebsiella/, accessed on 10 October 2025) for allele assignment and K-type determination.

### 4.9. Statistical Analysis

To evaluate differences in polymyxin B resistance among isolates from different sources, a Chi-square test of independence was performed. Pairwise comparisons of resistance proportions were subsequently conducted using two-proportion Z-tests with Bonferroni correction for multiple testing. Pairwise comparisons of resistance rates among sources (clinical, bed rails and wastewater) were performed for each antibiotic using Fisher’s Exact Test, with Bonferroni correction applied for multiple testing. Fisher’s Exact Test was also used to perform pairwise comparisons of resistance rates among antibiotics and identify significant differences in antimicrobial susceptibility rates within each source. Isolates from the stream water source were excluded from the statistical analysis due to the low number of samples (*n* = 4), which could compromise the reliability of the comparisons. The associations between biofilm-forming categories and isolate sources and polymyxin B resistance were assessed using a Chi-square test of independence. All statistical analyses were conducted using Python (version 3.x), with the SciPy and statsmodels libraries, assisted by ChatGPT 4.0. A significance level of *p* < 0.05 was adopted for all tests. To visualize proportional distributions, stacked bar charts were generated using the ggplot2 package in R (v4.5.0). These charts displayed the distributions of resistance profiles, biofilm formation, and meropenem MIC categories, normalized to 100% for each source, with percentage values annotated. Furthermore, the relationship between K-type and source was illustrated using a Circos plot created with the circlize package in R (v4.5.0).

## 5. Conclusions

This study provides an integrated overview of carbapenem-resistant *K. pneumoniae* from clinical and hospital-associated sources, with the inclusion of a few environmental isolates under a One Health perspective. Most isolates exhibited extensively drug-resistant (XDR) profiles, with residual susceptibility restricted to a few therapeutic options such as amikacin, gentamicin, and ceftazidime–avibactam. The near-universal ability to form biofilms observed among isolates supports their persistence on inanimate surfaces and their potential to sustain horizontal gene transfer. Capsular typing revealed a predominance of specific K types—mainly K64, K24, K173, and K50—detected across different origins, suggesting the persistence of adaptable lineages under variable selective pressures. Together, these results provide complementary insights into the phenotypic and structural features associated with the persistence of carbapenem-resistant *K. pneumoniae* in healthcare-related environments, supporting continued monitoring of high-risk lineages and their resistance determinants.

## Data Availability

All datasets are provided in the Supplementary Information.

## Supplementary Materials

The following supporting information can be downloaded at https://www.mdpi.com/article/10.3390/antibiotics14111140/s1: Table S1: Complete dataset of *Klebsiella* spp. isolates, including identification, antimicrobial resistance profiles, biofilm classification, and capsular typing.

## Abbreviations

The following abbreviations are used in this manuscript:

AMR	Antimicrobial Resistance
AMRO	Americas Regional Office
ARG	Antibiotic resistance gene
ATCC	American Type Culture Collection
BigDye	BigDye Terminator Sequencing Chemistry
CMY-2	Cephamycinase type 2
CRE	Carbapenem-resistant enterobacteria
CRKP	Carbapenem-resistant *Klebsiella pneumoniae*
CZA	Ceftazidime–avibactam
DHA-1	Dhahran Hospital AmpC type 1
ESBL	Extended-spectrum β-lactamase
EUCAST	European Committee on Antimicrobial Susceptibility Testing
Fiocruz	Fundação Oswaldo Cruz
*gyr*A	DNA gyrase subunit A gene
HAI	Healthcare-Associated Infection
HGT	Horizontal Gene Transfer
ICU	Intensive Care Unit
INCQS	Instituto Nacional de Controle de Qualidade em Saúde
KPC	*Klebsiella pneumoniae* carbapenemase
K-type	Capsular type
L-Ara4N	4-Amino-4-Deoxy-L-Arabinose
LPS	Lipopolysaccharide
MALDI-TOF	Matrix-Assisted Laser Desorption/Ionization—Time of Flight
*mcr*-1	Mobilized colistin resistance gene-1
MDR	Multi-drug-resistant
MGE	Mobile Genetic Element
MIC	Minimum inhibitory concentration
MTB_autoX	An automated acquisition method used in MALDI-TOF for microbial identification, ensuring standardized spectral data collection
NDM	New Delhi metallo-β-lactamase
OD	Optical Density
OXA-48	Oxacillinase-48
PAHO	Pan American Health Organization
*par*C	DNA topoisomerase IV subunit C gene
PBS	Phosphate-Buffered Saline
PDR	Pan-drug-resistant
PhoPQ	Two-Component Regulatory System PhoPQ
PmrAB	Two-Component Regulatory System PmrAB
POLB	Polymyxin B
PPT	Piperacillin–tazobactam
*qnr*	Quinolone resistance gene
ReLAVRA	Latin American Network for Antimicrobial Resistance Surveillance
SciPy	Scientific Python
STX	Sulfamethoxazole-trimethoprim
TAE	Tris-Acetate-EDTA Buffer
TSI	Triple Sugar Iron
XDR	Extensively drug-resistant
